# Low BASDAI score alone is not a good predictor of anti-tumor necrosis factor treatment efficacy in ankylosing spondylitis: a retrospective cohort study

**DOI:** 10.1186/s12891-020-03941-8

**Published:** 2021-02-04

**Authors:** Bora Nam, Bon San Koo, Tae-Han Lee, Ji-Hui Shin, Jin-Ju Kim, Seunghun Lee, Kyung Bin Joo, Tae-Hwan Kim

**Affiliations:** 1 Department of Rheumatology, Hanyang University Hospital for Rheumatic Diseases, 222-1 Wangsimni-ro, Seongdong-gu, Seoul, 04763 Republic of Korea; 2 Department of Rheumatology, Inje University Seoul Paik Hospital, Inje University College of Medicine, Seoul, South Korea; 3 Medical Affairs, AbbVie Ltd., Seoul, South Korea; 4 Department of Radiology, Hanyang University Hospital for Rheumatic Diseases, Seoul, South Korea

**Keywords:** Ankylosing spondylitis, Disease activity, Ankylosing spondylitis disease activity score (ASDAS), Bath Ankylosing spondylitis disease activity index (BASDAI), Anti-tumor necrosis factor (anti-TNF)

## Abstract

**Background:**

The purpose of this study was to determine the prevalence of high disease activity as measured using the Ankylosing Spondylitis Disease Activity Score (ASDAS) in ankylosing spondylitis (AS) patients who nonetheless have low Bath Ankylosing Spondylitis Disease Activity Index (BASDAI) scores after anti-tumor necrosis factor (TNF) treatment. Its clinical impact on anti-TNF survival was also investigated.

**Methods:**

We conducted a single-centre retrospective cohort study of AS patients having low BASDAI scores (< 4) and available ASDAS-C-reactive protein (CRP) data after 3 months of first-line anti-TNF treatment. Patients were grouped into high-ASDAS (≥ 2.1) and low-ASDAS (< 2.1) groups according to the ASDAS-CRP after 3 months of anti-TNF treatment. Their characteristics were compared. And survival analyses were carried out using Kaplan–Meier curves and log-rank test with the event being discontinuation of anti-TNF treatment due to lack/loss of efficacy.

**Results:**

Among 116 AS patients with low BASDAI scores after 3 months of anti-TNF treatment, 38.8% were grouped into the high-ASDAS group. The high-ASDAS group tended to have greater disease activity after 9 months of treatment (BASDAI 2.9 ± 1.1 vs. 2.3 ± 1.4, *p*=0.007; ASDAS-CRP 1.8 ± 0.6 vs. 1.5 ± 0.7, *p*=0.079; proportion of high ASDAS-CRP 27.8% vs. 13.8%, *p*=0.094) and greater risk of discontinuing anti-TNF treatment due to lack/loss of efficacy than the low-ASDAS group (*p*=0.011).

**Conclusions:**

A relatively high proportion of AS patients with low BASDAI scores had high ASDAS-CRP. These low-BASDAI/high-ASDAS-CRP patients also had a greater risk for discontinuation of anti-TNF treatment due to low/lack of efficacy than the low-ASDAS group. The use of the ASDAS-CRP alone or in addition to the BASDAI may improve the assessment of AS patients treated with anti-TNF agents.

## Background

Ankylosing spondylitis (AS) is characterised by inflammatory back pain which can lead to spinal structural damage. In addition, AS may also involve the peripheral joints, eyes, skin, and the intestinal systems. Non-steroidal anti-inflammatory drugs are typically the primary treatment option for AS, although anti-tumor necrosis factor (anti-TNF) agents have emerged as a novel alternative treatment [1]. Unlike other diseases, the severity of AS disease cannot be assessed using simple or direct “gold standard” measures, as is the case for glycosylated haemoglobin in diabetes mellitus and blood pressure in hypertension, due to the range and complexity of AS symptoms [2]. Although, several attempts have been made to develop a novel biomarker of AS disease activity and some interesting biomarkers have been identified [3, 4], composite scoring systems, namely the Bath Ankylosing Spondylitis Disease Activity Index (BASDAI) and/or Ankylosing Spondylitis Disease Activity Score (ASDAS) have been mainly applied when assessing AS disease activity [5, 6].

The BASDAI contains six questions addressing fatigue, back pain, peripheral joint pain/swelling, enthesitis, and morning stiffness [5]. Similar to the BASDAI, the ASDAS includes self-reported indices of back pain, duration of morning stiffness, peripheral joint pain/swelling, and patient global assessment of disease activity; however, the ASDAS also includes laboratory test results, such as the C-reactive protein (CRP) or erythrocyte sedimentation rate (ESR), and each parameter is weighted, not simply added up as for the BASDAI [6]. Although ASDAS might be affected infections or conditions influencing inflammatory markers, the ASDAS has been considered more objective than the BASDAI and was recently recommended as the “target” scale for AS treatment [7, 8]. Nevertheless, the BASDAI is still widely used because it is simpler and less time-consuming than the ASDAS. Moreover, the BASDAI is used to measure disease activity to initiate or maintain TNF inhibitor therapy in routine clinical practice and is a standard parameter when the cost of anti-TNF therapy for Korean AS patients is covered under the Korean National Health Insurance Service [9].

In some AS patients with low disease activity as measured by the BASDAI, the patient may nonetheless complain of pain or have elevated inflammatory markers. In these cases, assessing disease activity with other measurement tools such as the ASDAS can be helpful. In some such cases, we may find that some patients have both high ASDAS and low BASDAI scores at the same time.

Inaccurate measurement of disease activity can lead to inappropriate treatment and/or medication of AS patients. Therefore, the aim of this study was to determine the prevalence of high disease activity as measured using the ASDAS-CRP, in AS patients who nonetheless have low BASDAI scores after anti-TNF treatment. The clinical impact of this discordance in disease activity on anti-TNF survival was also investigated.

## Methods

### Patient recruitment

We conducted a retrospective cohort study of AS patients who began first-line anti-TNF treatment between January 2012 and December 2016 in a single tertiary referral hospital. All patients satisfied the 1984 modified New York criteria for the classification of AS [10]. Only patients having both BASDAI and ASDAS-CRP data available after 3 months (6 weeks for Infliximab) of anti-TNF treatment were included. Patients with high BASDAI scores (≥ 4) after 3 months (6 weeks for Infliximab) of anti-TNF treatment were excluded. Patients with other conditions affecting CRP, such as inflammatory bowel disease or infectious disease, were also excluded.

Enrolled patients were categorised into either the high-ASDAS (ASDAS-CRP ≥ 2.1) or low-ASDAS (ASDAS-CRP < 2.1) groups according to the ASDAS-CRP assessment completed after 3 months (6 weeks for infliximab) of anti-TNF treatment.

This study was performed according to the guidelines of the Helsinki Declaration and approved by the institutional review board of Hanyang University Hospital (IRB file No. HYUH 2019–09-016). Patient consent to participate was waived as this is a retrospective study.

### Data collection

We obtained demographic (age and sex) and disease-related clinical data (disease duration [duration since first AS-specific symptoms], history of uveitis or psoriasis, peripheral joint involvement, human leukocyte antigen [HLA]-B27 positivity, and type of anti-TNF agent) by medical chart review. The serum concentration of CRP and ESR, BASDAI score, and ASDAS-CRP were obtained at 0 month, 3 months (6 weeks for Infliximab), and 9 months after initiation of anti-TNF treatment. In addition, radiographs obtained within 2 years of the index date were scored using the modified Stoke Ankylosing Spondylitis Spinal Score (mSASSS).

The index date was defined as the date on which the BASDAI and ASDAS-CRP were measured 3 months (6 weeks for Infliximab) after initiation of the first-line anti-TNF treatment. All enrolled patients were observed until the time of their last visit, discontinuation of anti-TNF treatment, or December 2019.

The reason for discontinuation of anti-TNF treatment was collected as reported by the treating rheumatologist for the following pre-specified and mutually exclusive categories: clinical remission, lack/loss of efficacy, and adverse events.

### Statistical analyses

The demographic and clinical characteristics of the patients were summarised in a descriptive analysis. To compare the characteristics of the high-ASDAS and low-ASDAS groups, Student’s t tests were used for normally distributed data or a Mann-Whitney U test for non-normally-distributed data was used for continuous variables; dichotomous variables were analysed using the Chi-square test.

Survival analyses were carried out using Kaplan–Meier curves with the event being discontinuation of anti-TNF treatment due to lack/loss of efficacy. Patients discontinuing anti-TNF treatment due to clinical remission or adverse events were not included in the statistical analyses. In addition, the survival curves of the two groups were compared using the log-rank test.

To identify possible risk factors associated with high ASDAS-CRP after 3 months (6 weeks for infliximab) of anti-TNF treatment, multivariable logistic regression analyses were completed. Variables with *p*-values < 0.2 in the univariable model were advanced to the multivariable logistic regression models. In addition, age was retained as a parameter in the multivariable models.

All analyses were performed using SPSS® software (version 26.0; SPSS Inc., IL, USA). *P*-values < 0.05 were considered statistically significant.

## Results

### Clinical characteristics of patients

**Fig. 1:**
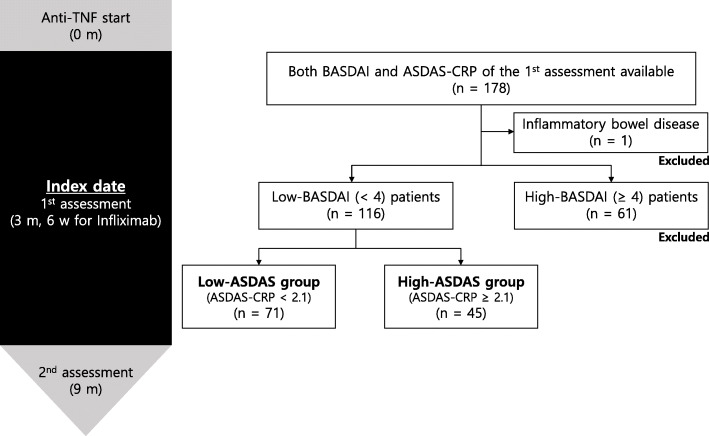
Flow chart of patient selection. BASDAI, Bath Ankylosing Spondylitis Disease Activity Index; ASDAS, Ankylosing Spondylitis Disease Activity Score, CRP, C-reactive protein; TNF, tumor necrosis factor

A total of 116 AS patients were included in this study, with 41 patients receiving adalimumab, 34 etanercept, 27 infliximab, and 14 golimumab (Fig. 1). The demographic and clinical characteristics of the patients at enrolment are shown in Table 1. The median age of the patients was 33.9 (26.7–40.3) years and 94% were male. The median disease duration was 10.8 (5.3–15.5) years. With regard to disease activity, the mean BASDAI and ASDAS-CRP values were 2.3 ± 0.9 and 2.0 ± 0.5, and the median ESR and CRP concentration were 3.0 (2.0–6.0) mm/hr. and 0.2 (0.2–0.2) mg/dL, respectively. In addition, the median mSASSS was 9.0 (6.0–25.9).

**Table 1:** Demographic characteristics of the study population

	Variables (*n*=116)
Age at enrolment, years	33.9 (26.7–40.3)
Male sex	109 (94)
Disease duration, years	10.8 (5.3–15.5)
Follow-up duration, years	6.4 (4.5–7.1)
HLA B27 positivity	115 (99.1)
Peripheral joint involvement	52 (44.8)
History of psoriasis	2 (1.7)
History of uveitis	35 (30.2)
mSASSS	9.0 (6.0–25.9)
BASDAI	2.3 ± 0.9
ASDAS-CRP	2.0 ± 0.5
High ASDAS-CRP (≥ 2.1)	45 (38.8)
ESR, mm/hr. (normal ≤ 20)	3.0 (2.0–6.0)
CRP, mg/dL (normal ≤ 0.3)	0.2 (0.2–0.2)
Use of anti-TNF agents	
Adalimumab	41 (35.3)
Etanercept	34 (29.3)
Infliximab	27 (23.3)
Golimumab	14 (12.1)
Discontinuation of anti-TNF agent during follow up	13 (11.2)
Lack/loss of efficacy	9 (69.2)
Clinical remission	3 (23.1)
Adverse event	1 (7.7)

Numerical quantitative data were presented as the “mean ± SD” or “median (IQR)” and categorical data were presented as the “frequency (%)”

*SD* standard deviation, *IQR* interquartile range, *HLA B27* human leukocyte antigen B27, *mSASSS* modified Stoke Ankylosing Spondylitis Spinal Score, *BASDAI* Bath Ankylosing Spondylitis Disease Activity Index, *ASDAS* Ankylosing Spondylitis Disease Activity Score, *CRP* C-reactive protein, *ESR* erythrocyte sedimentation rate, *TNF* tumor necrosis factor

During a median follow-up duration of 6.4 (4.5–7.1) years, 14 patients (12.1%) discontinued their anti-TNF agents: 9 (69.2%) due to lack/loss of efficacy, 3 (23.1%) due to clinical remission, and 1 (7.7%) due to an adverse event.

### Prevalence and characteristic of AS patients with high ASDAS-CRP after anti-TNF treatment

Among the total patient population, 38.8% of the patients were categorised into the high-ASDAS group. The patients in the high-ASDAS group had significantly greater BASDAI scores (3.0 ± 0.6 vs, 1.8 ± 0.8, *p* < 0.001), ASDAS-CRP (2.5 ± 0.3 vs. 1.7 ± 0.2, *p* < 0.001), and ESR (5.0 [2.0–11.5] mm/hr. vs. 2.0 [2.0–5.0] mm/hr., *p*=0.003) measurements than those of the low-ASDAS group. The median CRP concentration was comparable between the two groups (0.2 [0.2–0.3] mg/dL vs. 0.2 [0.2–0.2] mg/dL, *p*=0.243); however, the percentage of patients with abnormal CRP concentrations was greater in the high-ASDAS group than the low-ASDAS group (17.8% vs. 1.4%, *p*=0.001).

Initial disease activity indices, including the BASDAI, ASDAS-CRP, and serum concentration of ESR and CRP, were comparable across the two groups. However, patients in the high-ASDAS group still had significantly greater BASDAI scores at 9 months (2.9 ± 1.1 vs. 2.3 ± 1.4, *p*=0.007) than patients of low-ASDAS group. The mean ASDAS-CRP value and proportion of patients with high ASDAS-CRP assessed at 9 months were greater in patients in the high-ASDAS group, but these results were not statistically significant (1.8 ± 0.6 vs. 1.5 ± 0.7, *p*=0.079; 27.8% vs. 13.8%, *p*=0.094).

A total of 9 patients discontinued anti-TNF treatment due to lack/loss of efficacy, of which 7 cases occurred in the high-ASDAS group and 2 in the low-ASDAS group. All cases of discontinuation due to clinical remission occurred in the low-ASDAS group (Table 2).

**Table 2:** Comparisons of demographic and clinical characteristics between the high-ASDAS and low-ASDAS groups

	High-ASDAS group (*n*=45)	Low-ASDAS group (*n*=71)	*p*-value
Age, years	35.0 (30.2–40.4)	33.1 (25.6–40.3)	0.222
Male sex,	40 (88.9)	69 (97.2)	0.068
Disease duration, years	11.1 (6.1–15.3)	9.8 (4.6–15.8)	0.465
Follow-up duration, years	6.4 (4.0–6.9)	6.5 (4.6–7.2)	0.289
HLA B27 positivity	44 (97.8)	71 (100)	0.207
Peripheral joint involvement	22 (48.9)	30 (42.3)	0.484
History of psoriasis	1 (2.2)	1 (1.4)	0.751
History of uveitis	13 (28.9)	22 (31.0)	0.811
mSASSS	12.0 (6.0–26.7)	8.0 (6.0–23.7)	0.256
BASDAI	3.0 ± 0.6	1.8 ± 0.8	< 0.001
ASDAS-CRP	2.5 ± 0.3	1.7 ± 0.2	< 0.001
ESR, mm/hr	5.0 (2.0–11.5)	2.0 (2.0–5.0)	0.003
Abnormal ESR	7 (15.6)	2 (2.8)	0.012
CRP, mg/dL	0.2 (0.2–0.3)	0.2 (0.2–0.2)	0.243
Abnormal CRP	8 (17.8)	1 (1.4)	0.001
Initial BASDAI (0 month)	6.7 ± 1.3 (n=45)	6.7 ± 1.5 (n=71)	0.768
Initial ASDAS-CRP (0 month)	3.9 ± 0.8 (*n*=35)	4.0 ± 1.0 (*n*=57)	0.471
Initial ESR (0 month), mm/hr	31.5 (17.8–55.0) (n=43)	38.0 (21.0–76.0) (n=70)	0.331
Initial CRP (0 month), mg/dL	1.6 (0.9–3.4) (*n*=43)	1.9 (0.2–4.7) (*n*=70)	0.562
Follow-up BASDAI (9 months)	2.9 ± 1.1 (n=45)	2.3 ± 1.4 (n=71)	0.007
Follow-up ASDAS-CRP (9 months)	1.8 ± 0.6 (*n*=36)	1.5 ± 0.7 (*n*=58)	0.079
High ASDAS-CRP (9 months)	10 (27.8%)	8 (13.8%)	0.094
Follow-up ESR (9 months), mm/hr	5.0 (2.0–11.0) (n=39)	2.0 (2.0–7.0) (*n*=64)	0.299
Follow-up CRP (9 months), mg/dL	0.2 (0.2–0.2) (*n*=39)	0.2 (0.2–0.2) (*n*=65)	0.795
Discontinuation of anti-TNF agent	8 (17.8)	5 (7.1)	0.022
Lack/loss of efficacy	7 (87.5)	2 (40)	
Clinical remission	0 (0)	3 (60)	
Adverse event	1 (12.5)	0 (0)	

Numerical quantitative data were presented as the “mean ± SD” or “median (IQR)” and categorical data were presented as the “frequency (%)”

*SD* standard deviation, *IQR* interquartile range, *HLA B27* human leukocyte antigen B27, *mSASSS* modified Stoke Ankylosing Spondylitis Spine Score, *BASDAI* Bath Ankylosing Spondylitis Disease Activity Index, *ASDAS* Ankylosing Spondylitis Disease Activity Score, *CRP* C-reactive protein, *ESR* erythrocyte sedimentation rate, *TNF* Tumor necrosis factor

### Factors associated with high ASDAS-CRP after anti-TNF treatment

To identify factors associated with a high ASDAS-CRP result after 3 months (6 weeks for infliximab) of anti-TNF treatment, we used univariable and multivariable logistic regression analyses. Female sex was shown to be a contributor for a high ASDAS-CRP (odds ratio [OR] 5.92, 95% CI 1.05–33.46, *p*=0.044) (Table 3).

**Table 3:** Factors associated with greater assessed ASDAS-CRP after 3 months (12 weeks) of anti-TNF treatment (*n*=92)

	Univariable analysis		Multivariable analysis	
	OR	*p*-value	OR	*p*-value
Age^a^	1.26 (0.82–1.96)	0.296	1.18 (0.73–1.90)	0.509
Female sex	4.31 (0.80–23.27)	0.089	5.92 (1.05–33.46)	0.044
Disease duration^b^	1.18 (0.75–1.85)	0.469		
Initial BASDAI (0 month)	0.96 (0.73–1.26)	0.772		
Initial ASDAS-CRP (0 month)	0.85 (0.54–1.35)	0.493		
High mSASSS (> median)	1.81 (0.85–3.85)	0.123	1.95 (0.85–4.45)	0.113
Peripheral arthritis	1.31 (0.62–2.77)	0.484		
Uveitis	0.91 (0.40–2.05)	0.811		
Psoriasis	1.57 (0.10–25.72)	0.753		

^a^Root-transformed value was used

^b^Log-transformed value was used

*ASDAS* Ankylosing Spondylitis Disease Activity Score, *CRP* C-reactive protein, *TNF* tumor necrosis factor, *OR* odds ratio, *BASDAI* Bath Ankylosing Spondylitis Disease Activity Index, *mSASSS* modified Stoke Ankylosing Spondylitis Spine Score

### Retention rates for anti-TNF agents according to disease activity states

**Fig. 2:**
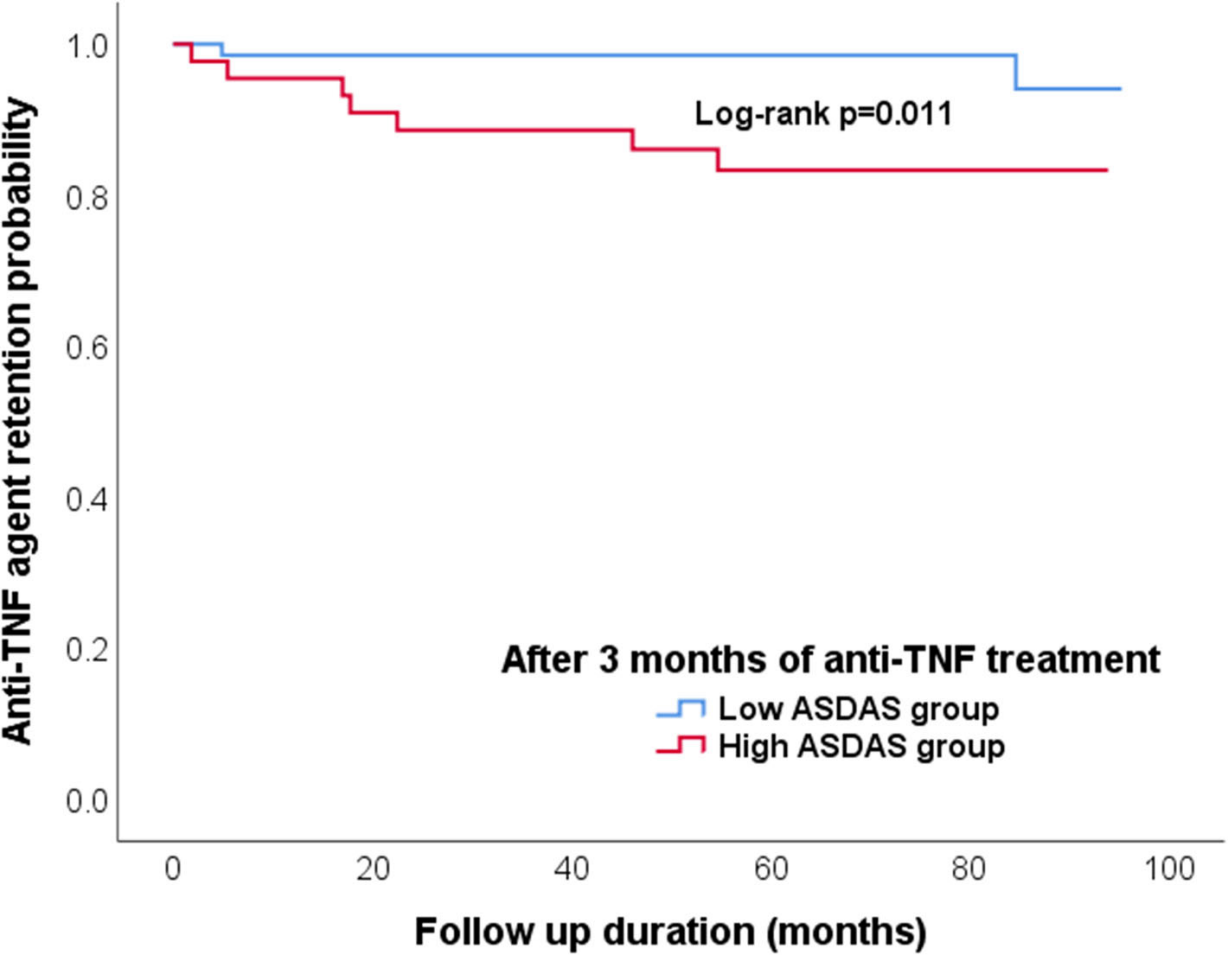
Comparison of the anti-TNF agent retention rates according to ASDAS-CRP. TNF; tumor necrosis factor; ASDAS, The Ankylosing Spondylitis Disease Activity Score, CRP, C-reactive protein

Kaplan-Meier curves for time to discontinuation due to lack/loss of efficacy for low and high ASDAS group are shown in Fig. 2. The survival of high-ASDAS group patients receiving anti-TNF agents was significantly lower than in those of low ASDAS group (log-rank *p*=0.011).

The median time to discontinuation of treatment among patients of the high-ASDAS group was 19.7 (5.5–46.1) months, while the duration of therapy for the 2 cases in the low-ASDAS group who discontinued treatment was 4.9 months and 44.8 months.

## Discussion

In this retrospective cohort study, we found that 38.8% of patients with low BASDAI scores (< 4) after 3 months (6 weeks for infliximab) of anti-TNF treatment had high disease activity according to ASDAS-CRP assessment (≥ 2.1). These patients tended to have more severe disease activity after 9 months of anti-TNF treatment and greater risk of discontinuation of anti-TNF treatment than those with low disease activity according to both ASDAS-CRP and BASDAI scores. Female sex was also associated with greater ASDAS-CRP values after 3 months (6 weeks for infliximab) of anti-TNF treatment.

There have been few previous studies address in AS disease activity as measured by different assessment systems; however, some studies investigating AS patients treated with anti-TNF agents have provided valuable data. Consistent with our results, one previous study indicated that 36.9% (210/568 patients) of patients with low BASDAI scores (< 4) had high disease activity as measured by the ASDAS-CRP (≥ 2.1) [11]. Moreover, even higher percentages of patients having high ASDAS-CRP (≥ 2.1) among those having low BASDA score (< 4) were shown in two other studies; 62.1% (64/103 patients) and 65.8% (48/73 patients) [12, 13].

Assessing more patients into worse conditions cannot be simply judged as better measurement. Interestingly, we found differences in prognosis between patient groups based on the ASDAS-CRP assessment. Patients with high ASDAS-CRP appeared to have higher disease activity at their follow-up visits despite anti-TNF treatment. Similar results were obtained in previous studies in which the immediate response to the first anti-TNF agent was shown to largely determine the long-term prognosis [14]. In addition, the anti-TNF agent retention rate was significantly lower in AS patients with high ASDAS-CRP results than among patients with low ASDAS-CRP results, although all would be considered to have low disease activity based on their BASDAI score.

“Treat-to-target (T2T)” is a major principle in treating many diseases, such as hypertension, diabetes mellitus, and dyslipidemia. In the past decade, promotion of T2T has spread to inflammatory rheumatic diseases, including rheumatoid arthritis, spondyloarthritis (SpA), systemic lupus erythematosus, and gout [15–18]. After determining the desired target values, the objectives of T2T are to ensure accurate and reliable disease assessment and more reasonable treatment decisions (i.e., whether treat or not treat and switch or maintain medications), and to thereby obtain better outcomes. In view of the potential adverse effects and financial burden of anti-TNF treatment, a T2T approach should be emphasised in AS patients treated with anti-TNF agents.

Several attempts have been made to compare the discriminatory capacity of the ASDAS with that of BASDAI in AS patients treated anti-TNF agents. One previous study investigating eligibility criteria for initiation of anti-TNF treatment in AS patients showed that an ASDAS-CRP result ≥ 2.1 is associated with a greater likelihood of improvement than a high BASDAI score (≥ 4), but the patients who had both ASDAS-CRP ≥ 2.1 and BASDAI ≥ 4 showed the greatest improvement [12]. Similarly, from another prospective cohort study, applying the ASDAS definition of high disease activity leads to more axial SpA patients being selected to start anti-TNF treatment and these ‘captured’ patients have a greater likelihood of known predictors of good response to anti-TNF than traditional BASDAI definition (≥ 4) [13]. One study including patients with AS and undifferentiated SpA treated with etanercept or infliximab revealed that the discriminatory ability of the ASDAS outperformed that of the BASDAI, patient global score, ESR, CRP, or acute inflammation score as assessed by MRI [19]. In addition, a previous study investigating the levels and changes of 10 biomarkers in axial SpA patients during anti-TNF treatment revealed that the ASDAS-CRP was more strongly associated with these biomarkers than the BASDAI [20].

Moreover, the ASDAS seems to better predict the patient prognosis than the BASDAI. One prospective study of 2274 AS patients newly treated with anti-TNF agents indicated that a low ASDAS-CRP (< 2.1) result after 3 months of anti-TNF treatment predicted the risk of future non-disability better than a low BASDAI (< 4) result and low CRP concentration (< 5 mg/L) [14]. A 12-year longitudinal study using data from the OASIS cohort revealed that models including the ASDAS-CRP as the disease activity measure fitted the data better than models including the BASDAI, CRP, or both the BASDAI and CRP. In addition, an increase of one ASDAS-CRP unit led to an increase of 0.72 mSASSS units/2 years [21]. Similarly, in another study from the German SpA inception cohort, radiographic spinal progression was found to be associated with the time-averaged ASDAS-CRP (mSASSS progression by over 2 points/2 years: OR 1.80, 95% CI 1.04–3.13; syndesmophyte formation/progression: OR 2.45, 95% CI 1.26 to 4.77) [22]. However, no associations between radiographic progression and BASDAI results have been reported [22, 23].

Female sex was associated with high ASDAS-CRP results after 3 months (6 weeks for infliximab) of anti-TNF treatment, which may be attributed to the very small population of female patients in our study population: only five patients were female in the high-ASDAS group, while only 2 patients were female in the low-ASDAS group. On the other hand, several researchers have reported that females were less likely to respond to anti-TNF treatment and were more likely to discontinue anti-TNF treatment early based on data from registries of rheumatic disease including AS [24–29]. However, to date, there has been no clear determination of the basis of sex-dependent differences in anti-TNF response and survival, and further study is therefore needed to clarify the relationships between anti-TNF treatment and sex.

The present study has some limitations. First, we only focused ASDAS-CRP although there are available novel activity indices for AS such as the BASDAS formula, similar to the ASDAS-CRP but uses only the BASDAI and CRP, and simplified ASDAS [30, 31]. Measuring ASDAS-CRP is not yet routine in clinical practice. Therefore, the size of our study population was relatively small and selection bias may have occurred due to the retrospective nature of the study; some patients suspected of high disease activity despite of low BASDAI might be assessed with ASDAS-CRP. Second, the number of female patients was small. Therefore, some results need to be interpreted with some caution. It might be explained by relatively high proportion of female patients having high BASDAI (≥ 4) after 3 months of anti-TNF treatment (12 females among total 61 patients) and consequently being excluded. One of possible explanations of it might be characteristics of female AS patients; several studies have shown that female AS patients tended to report high BASAI score compared to males [32–35]. Or it might be associated to which we suggested in our study; females were less likely to respond to anti-TNF treatment. Third, we only considered disease activity when comparing survival during anti-TNF treatment, and did not consider the type of anti-TNF agent, doses, or dosing intervals in the analyses. Despite these limitations, the present study provides valuable real-world data obtained from AS patients treated anti-TNF agents and focused on discordances in disease activity that have rarely been assessed previously. We found a benefit of assessing the ASDAS-CRP with respect to prognosis and drug survival during treatment with anti-TNF agents. Although the applicability of ASDAS-CRP might vary across different regions since the measuring ASDAS-CRP needs laboratory tests and the calculator of digital system [31].

To conclude, a low BASDAI score after anti-TNF treatment may not be as meaningful as previously thought. About 40% of low-BASDAI patients may have high AS disease activity according to an ASDAS-CRP assessment and be at higher risk of discontinuation of anti-TNF treatment due to lack/loss of effectiveness. In addition, all cases in which anti-TNF treatment was discontinued due to clinical remission were in the low-ASDAS group. In consideration of our results and the great capacity of the ASDAS shown in previous studies, we suggest the use of the ASDAS-CRP alone or in addition to the BASDAI when assessing disease activity in AS patients treated with anti-TNF agents.

## Data Availability

The datasets are available from the corresponding author upon reasonable request.
